# A Leukemic Target with a Thousand Faces: The Mitochondria

**DOI:** 10.3390/ijms241713069

**Published:** 2023-08-22

**Authors:** Beatrice Maffeo, Cristina Panuzzo, Amedeo Moraca, Daniela Cilloni

**Affiliations:** Department of Clinical and Biological Sciences, University of Turin, 10043 Orbassano, Italy; beatrice.maffeo@unito.it (B.M.); amedeo.moraca@unito.it (A.M.); daniela.cilloni@unito.it (D.C.)

**Keywords:** AML, personalized therapies, AML–LSC, AML blasts, mitochondria, metabolism, Venetoclax

## Abstract

In the era of personalized medicine greatly improved by molecular diagnosis and tailor-made therapies, the survival rate of acute myeloid leukemia (AML) at 5 years remains unfortunately low. Indeed, the high heterogeneity of AML clones with distinct metabolic and molecular profiles allows them to survive the chemotherapy-induced changes, thus leading to resistance, clonal evolution, and relapse. Moreover, leukemic stem cells (LSCs), the quiescent reservoir of residual disease, can persist for a long time and activate the recurrence of disease, supported by significant metabolic differences compared to AML blasts. All these points highlight the relevance to develop combination therapies, including metabolism inhibitors to improve treatment efficacy. In this review, we summarized the metabolic differences in AML blasts and LSCs, the molecular pathways related to mitochondria and metabolism are druggable and targeted in leukemia therapies, with a distinct interest for Venetoclax, which has revolutionized the therapeutic paradigms of several leukemia subtype, unfit for intensive treatment regimens.

## 1. Introduction

Acute myeloid leukemia (AML) is an aggressive disease that is still characterized by a dismal prognosis in a significant number of patients: the 5-year overall survival (OS) in the adult population is around 28%, but if patients over 65 are selected, OS is less than 10% [1]. Similarly, patients affected by high-risk AMLs have an OS below 20%, despite age [2,3].

Disappointingly, AML prognosis has not dramatically changed, even though the pharmacological armamentarium against AML has significantly increased over the last few years, currently including: (i) Classical chemotherapeutic agents: they are the mainstay of intensive therapy, but chemo-resistant clones are often selected, e.g., those harboring TP53 mutation or complex karyotypes. Moreover, chemotherapy generally spares leukemic stem cells (LSCs), leading to relapse; (ii) Mutation-specific agents, e.g., FMS-like tyrosine kinase 3 (FLT3) or isocitrate dehydrogenase 1/2 (IDH1/2) inhibitors [4,5]. While generally being well-tolerated, these drugs only address the mutation-carrying clone, quickly leading to clonal escape. (iii) BH3 mimetics, e.g., the Bcl-2 inhibitor Venetoclax [6,7]. While theoretically usable in every AML, its effectiveness in monotherapy is modest, and even in combination with hypomethylating agents, the duration of response is limited to few months. Resistant clones rapidly emerge, generally carrying anti-apoptotic mechanisms other than Bcl-2 (e.g., mutations in BAX (*BCL2* associated X), overexpression of MCL1 or other members of the Bcl-2 family, and TP53 mutations) [8]. Additional emerging strategies that target specific molecules necessary for leukemic cell survival are in developing. An example is represented by magrolimab, an anti-CD47 antibody, which enhances macrophage antileukemic activity [9].

Moreover, in the last years, much effort has been made to highlight metabolic differences between tumor cells and healthy cells, with particular attention to mitochondria [10]. The maintenance of a functional mitochondrial structure is fundamental for cell survival, and the dysregulation of mitochondrial function often precedes the malignant transformation of hematopoietic stem cells (HSCs) [11]. Furthermore, leukemia stem cells (LSCs), the main cause of disease relapse, have distinct metabolic properties involving mitochondria. Targeting these metabolic pathways could potentially lead to the eradication of LSCs and prevent relapse [12,13].

For all the above reasons, numerous therapies and ongoing clinical trials for AML treatment are concentrating on mitochondrial molecules [10] and have shown potential, including Venetoclax, an efficient Bcl-2 inhibitor, which acts on mitochondrial cristae structure and promotes mitochondrial apoptotic pathway [14] and other drugs directed against specific components of the electron transport chain (ETC) like Complexes I and III [15,16,17,18].

In summary, AML is still an urgent clinical need, and the scientific community is seeking a new approach to address the issue of clonal escape, which is particularly fostered by mono- and mutation-specific therapies and target LSCs, which are probably the quiescent reservoir promoting relapse and are generally resistant to chemotherapy.

An ideal approach should be a comprehensive, multi-targeted strategy: while singular agents could be insufficient or promote clonal escape, their combination would be lethal to leukemic cells. This concept is somehow similar to the so-called synthetic lethality in genetics. The relationship between cancer and metabolism is complex and bidirectional. However, oncogenes force and alter several metabolic pathways to support cancer progression, leading to metabolic addiction [19]. However, the dysregulation of metabolism can be directly tumorigenic, as for IDH1/2 mutations [20]. The idea to target cancer metabolism dates to the 1940s. Since then, classical antimetabolites (pyrimidine or purine analogues, anti-folate drugs) have represented a mainstay of cancer therapy.

## 2. Altered Metabolic Processes in AML LSCs

Cancer cells undergo a metabolic reprogramming, which has been extensively described, called the Warburg effect [21], which consists of increased glucose consumption due to the energy production based on fermentation of glucose to lactate regardless of oxygen availability. Leukemia cells present a unique metabolic signature and transcend the conventional Warburg effect [22,23]. In addition, leukemic cells display a dysregulation of the principal metabolic pathways, such as the mTOR and PI3K/AKT pathways with higher glucose uptake [24,25]. The first step of glycolysis involves the enzymes hexokinases, which catalyze the conversion of glucose to glucose-6-phosphate. Hexokinase II is shown to be frequently upregulated in cancer cells [26,27]. Even though targeting hexokinase II does not directly affect AML cells, the treatment can sensitize the cells to other drugs that affect mitochondria [28]. In addition, leukemic cells upregulate FAO through mitochondrial uncoupling [29] and glutaminolyisis [21]. Particularly, mutations in FLT3, which are highly prevalent in a newly diagnosed AML, have been correlated with increased glutaminolysis levels [30,31]. Finally, leukemic cells display a higher level of anabolic intermediates, such as the pentose phosphate pathway (PPP) and the citric acid cycle (CAC) [10], while 20% of AML patients present mutations in the IDH genes, which affect mitochondrial metabolism and is associated with poor prognoses [26].

**Figure 1:**
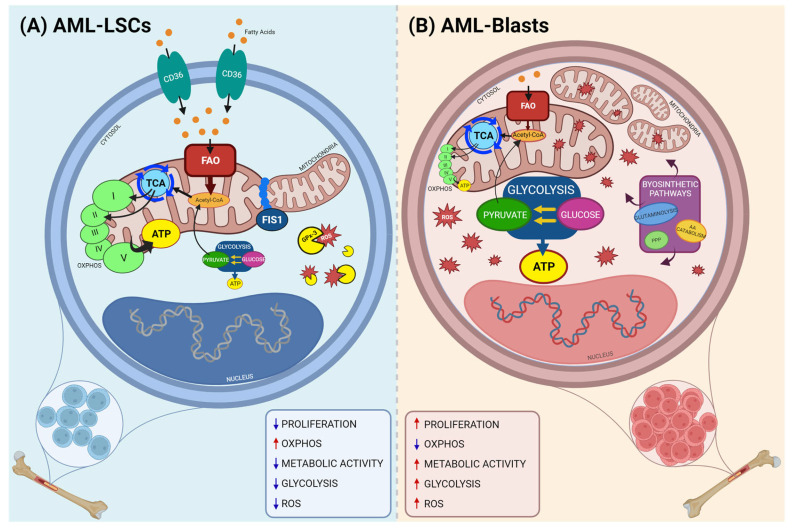
Metabolic differences between LSCs and blasts in AML. Graphic representation of metabolic differences between AML–LSCs cells and AML blasts. (**A**) AML–LSCs are dependent on OXPHOS, crucial for high ATP production, exhibit low ROS level and use amino acid and fatty acid metabolism to sustain the citric acid cycle. (**B**) On the contrary, AML blasts are more reliant on glycolysis for ATP production and have a higher metabolic activity. In addition, blasts have a higher number of mitochondria and a higher ROS level. Abbreviation: LSCs: leukemic stem cells, TCA: tricarboxylic acid; FAO: fatty acid oxidation; OXPHOS: oxidative phosphorylation; ATP: adenosine triphosphate; FIS1: mitochondrial fission 1 protein; GPx-3: Glutathione peroxidase 3; ROS: reactive oxygen species; AA: amino acids; PPP: pentose phosphate pathway; I–V: complexes of electron transport chain. Red and blue arrows in the boxes correspond to increase and decrease respectively. The figure is created with “https://biorender.com/ (accessed on 20 July 2023)”.

Myeloblasts are immature myeloid progenitor cells, which are located in both peripheral blood and bone marrow and are responsible for AML development. Myeloblasts tend to be intensively proliferative, thus generating a bulk of non-functional cells that compromises hematopoiesis and leads to the leukemic disease [32]. In addition to blasts, a population of leukemic stem cells (LSCs) reside in the bone marrow of AML patients, which is presently considered the main cause of relapse [12]. LSCs share many metabolic characteristics with normal hematopoietic stem cells (HSCs), including a low rate of division and replication [12]. However, LSCs show significant metabolic differences when compared to AML blasts (Figure 1). The most evident difference is that unlike AML blasts, LSCs divide slowly. Notably, their slower proliferative rate makes them less affected by conventional anti-proliferative treatment. Indeed, LSCs also represent a reservoir for the re-emergence of rapidly dividing myeloblasts with a crucial role in relapse and treatment resistance [10,12]. Numerous studies indicated a unique metabolic reprogramming in AML blasts, mainly based on high glycolytic activity [22]. However, LSCs have a metabolic profile that is active like healthy HSCs, thus challenging current AML treating strategies [13].

Consistent with higher glycolysis levels and elevated proliferative rate, a metabolic analysis on AML blasts showed increased levels of anabolic pathway precursors and high biosynthetic pathway activity, which is necessary to sustain cell growth and proliferation [24]. However, LSCs proliferate more slowly and rely on oxidative phosphorylation (OXPHOS) for ATP production [33,34]. A proteomic-based comparison has recently observed a consistently higher amount of several components of electron transport chain (ETC) Complexes I and V in LSCs compared to HSCs and blasts [35]. For the ETC to function, a regular supply of NADH and FADH2 is needed to provide electrons to oxygen, and the cofactor availability is ensured by tricarboxylic acid (TCA) cycle activity. Consistently, Jones et al. demonstrated that amino acids metabolism is a source of TCA cycle intermediates, and LSCs can use a wider source of TCA cycle substrates to fuel mitochondrial respiration than AML blasts [36,37]. LSCs also are reliant on different fuel sources than AML blasts, such as adipocytes. Indeed, AML LSCs preferentially locate in extramedullary adipose niches and overexpressed fatty acid transporter CD36 [38,39]. Moreover, LSCs show increased β-oxidation activity, suggesting that these cells are likely using adipocytes-derived fatty acids to fuel the TCA cycle and ETC [29].

Both AML LSCs and blasts have shown an increased mitochondrial content compared with healthy HSCs, nonetheless, concurrently, no increase respiratory functions have been observed, suggesting that the mitochondria could be less efficient [40,41]. The LSCs’ lower mitochondrial could be mediated by mitochondrial fission 1 protein (FIS1) clearance of dysfunctional mitochondria since mitochondrial fission mediated by FIS1 is necessary for LSC survival and Leukemia-initiating capacity [42]. In addition, reactive oxygen species are generated during mitochondrial oxidative metabolism and play a central role in cellular signaling [43,44]. Moreover, the overproduction of ROS is toxic to cells and particularly to LSCs, which can activate the metabolic-stress regulator AMP-activated protein kinase (AMPK), master regulator of FIS1, to modulate mitophagy activity and avoid AML LSCs damage. However, the inhibition of FIS1-mediated mitophagy induces myeloid differentiation, reduction in cell cycle activity, and loss of leukemic stem and progenitor cell potential [42]. In addition, AML LSCs display an increased expression of the ROS-scavenging enzyme glutathione peroxidese 3 (GPX-3) [45], while higher ROS levels are less toxic to AML blasts. In addition, AML LSCs display increased levels of mitochondrial transporters, such as mitochondrial carrier homolog 2 (MTCH2), which is a mitochondrial outer membrane protein insertase fundamental for pyruvate uptake into mitochondria and linked to LSCs survival and differentiation [46,47]. Besides MTCH2, numerous genes encoding for intermediates of pathways linked to mitochondrial transport are upregulated in AML LSCs, highlighting how mitochondrial are dynamic [37]. Lastly, ROSs represent a significant threat for most cellular types, including AML cells [48]. The accumulation of ROS beyond antioxidant defense capacity can promote cell death by activating both mitochondrial and cell pathways of apoptosis. Mitochondrial ROS production and accumulation leads to mitochondrial damages, including an alteration in mtDNA, disruptions in respiratory chain functions, and a loss of mitochondrial membrane potential that in turn impairs mitochondrial functions and promotes mitophagy [49,50]. Mitophagy is the selective degradation of mitochondria by autophagy, which is an intracellular lysosomal degradation pathway [51]. The two main regulators of mitophagy are PTEN-induced kinase 1 (PINK1) and the E3 ubiquitin ligase Parkin, which form the ubiquitin-dependent mitophagy pathway [52]. In normal conditions, PINK1 is imported in mitochondria and immediately clavated by resident proteases [53]. However, under stress conditions, PINK1 accumulates on the mitochondrial surface, phosphorylating itself and Parkin, thus allowing mitochondria to be recognized by autophagosome [50]. Otherwise, the ubiquitin-independent mitophagy pathway is regulated by proteins of the Bcl-2 family, including BNIP3, BNIP3L, and BCL2L13, as well as the anti-apoptotic proteins of the FKBP family, such as FK506-binding Protein 8 (FKBP8) [54,55].

All these data identify the significant metabolic differences between AML blasts and AML LSCs, highlighting the importance to develop new single or in combination therapies that target diverse metabolic pathways of both AML cell populations.

## 3. Mitochondrial Targets and Specific Therapies in AML

In recent times, several research groups identified distinctive changes in mitochondria within AML cells, which result in the deregulation of several metabolic processes. Mitochondrial dysfunction has been recognized as a significant factor in the development and progression of AML and is the basis of distinct metabolic properties between LSCs and AML blasts. In this regard, researchers are exploring mitochondrial features as potential target for developing specific therapies for AML. Here, we report some examples of therapies that specifically target metabolic pathways related to mitochondria and mitochondria itself at a date used either alone or in combination with other drugs (Table 1).

### 3.1. Tricarboxylic Acid (TCA) Cycle Inhibition

Like many other cancer cell types, LSCs primarily use the TCA cycle and OXPHOS to sustain proliferation [56]. Hence, a successful anti-leukemia strategy might be the targeting of enzymes involved in the flux of pyruvate into mitochondrial metabolism or TCA. IDHs are NADP+-dependent enzymes that catalyze the interconversion between isocitrate and α-ketoglutarate (α-KG) in the TCA cycle, and their mutations could drive the development of AML. IDH1 and IDH2 mutations have similar incidence and are mutually exclusive, resulting in the conversion of α-KG into the oncometabolite R-2-hydroxygluta (R-2-HG) [57,58]. R-2-HG interacts with α-KG-dependent enzymes and leads to DNA hypermethylation [59]. Ivosidenib (AG-120) and enasidenib (AG-221), are two inhibitors of IDH1 and IDH2, respectively, approved by the FDA (Food and Drug Administration) for AML treatment. These inhibitors prevent α-KG production and restore a normal DNA methylation profile [60]. Several clinical trials are ongoing on both IDH1 and IDH2 inhibitors [60]. Moreover, an abnormal accumulation of R-2-HG inhibits cytochrome c oxidase (COX), thus producing a stressing environment that in turn activates the pro-apoptotic proteins BAX and BAK. The anti-apoptotic Bcl-2 antagonizes BAK and BAX, promoting mutated IDH cells survival and a dependance on Bcl-2 [61]. Therefore, the Bcl-2 inhibitor Venetoclax could also act as an indirect IDH inhibitor for treatment of AML.

### 3.2. Electron Transport Chain (ETC) Inhibition

LSCs rely on OXPHOS for survival, and ETC is essential for OXPHOS process. ETC consists of four major multienzymatic complexes, and 13 out of 90 proteins of the ETC are encoded by mitochondrial DNA [15]. Poorer outcomes have been observed in AML patients with mutations in mitochondrial genes encoding for Complexes I, III, and IV of the ETC, suggesting that loss of proper functions worsens the disease. However, significant evidence demonstrated that the ETC complexes are suitable targets for therapeutic intervention, in particular, Complex I [16]. Mubritinib (TAK-165), a canonically inhibitor of the tyrosine kinase ERBB2 belonging to EGF receptor superfamily, showed strong anti-leukemic effects both in vitro and in vivo thanks to its ability to inhibit the function of Complex I [17]. In a combinatorial treatment, Venetoclax and azacitidine showed a synergistic effect on glutathionylation of succinate Dehydrogenase A (a component of Complex II) and decreases OXPHOS and energy production in patients with AML, killing both myeloblasts and LSCs [18]. In addition, Liyanage et al. demonstrated that ddC, a selective inhibitor of the mitochondrial DNA polymerase, inhibits ETC-related proteins, thus reducing the replication of mitochondrial DNA and inducing cell death [11].

### 3.3. Reactive Oxygen Species (ROS) Regulation

Reactive oxygen species (ROS) are biologically generated during cell metabolism, and their accumulation is dangerous for cell survival. For this reason, many clinical strategies are based on redox-based treatments. These compounds compromise the mitochondrial antioxidant system by promoting ROS accumulation [62]. AML clonal cells produce high levels of ROS, which is crucial for the bone marrow (BM) microenvironment and for leukemia progression [44,63]. Hence, two different therapeutic approaches targeting ROS production have been developed: the pro-oxidant approach and the antioxidant method. Chemotherapy alters the metabolic systems of leukemic cells, promoting ROS generation. Combination treatment with anthracycline and cytarabine increases ROS levels, inducing changes in the antioxidant system and, thus, programmed cell death [64]. Similarly, arsenic trioxide (ATO), a potent inhibitor of mitochondrial respiration and, consequently, a ROS inducer, is extensively used in association with all-trans retinoic acids (ATRA) to promote promyelocytic blast differentiation and apoptosis in promyelocytic leukemia (APL) [65,66]. In addition, ATO induces oxidative stress in AML cells, depolarization of the mitochondrial membrane, DNA damage, and, finally, apoptosis [67,68].

### 3.4. Amino Acid Metabolism Inhibition

During AML development, cells undergo changes in many aspects of their metabolism, including amino acid (AAs) metabolism, which largely relies on mitochondrial enzymes. These metabolic changes can lead to the identification of specific vulnerabilities of cancer cells and the development of several agents directed towards these specific targets [69]. Essential amino acids (EAAs) are not synthesized de novo or insufficiently synthesized by animal cells relative to metabolic needs, which is required by most tumor cells [70,71]. EAAs include tryptophan, phenylalanine, methionine, lysine, etc.

Methionine is an essential AA that plays a critical role in one-carbon metabolism and is a main source of intracellular methyl unit, which is essential for epigenetic modulation and RNA translation. Metabolomic profiling showed that AML patients display altered methionine abundance compared to healthy donors [72]. In myeloid leukemia cells, the isoenzymes of the methionine adenosyl transferase (MAT) family, also known as S-adenosylmethionine synthases, catalyze the conversion of methionine to S-adenosyl-methionine (SAM) and is correlated to poor prognosis [72,73]. In vitro studies on MAT and SAM inhibitors, PF-9366 and LLy-283, showed a reduction in the proliferation rate and viability of AML cell lines [74,75]. Moreover, methionine can be re-synthesized from homocysteine, which is obtained from the hydrolysis of SAH by S-adenosyl-homocysteine hydrolases (SAHH). In addition, 3-deazaadenosine (DZA) is a cyclic dinucleotide-based inhibitor of SAHH that promotes increased intracellular SAH levels and a decrease in overall methylation potential after treatment in primary AML blasts [76].

Similarly, tryptophane is an essential AA and has a main role in cancer immunity [77]. The heme-dependent endocellular enzymes tryptophan 2,3 dioxygenase (TDO) or indoleamine 2,3 dioxygenase (IDO) catalyze the oxidation of tryptophan to N-formyl kynurenine, which is then hydrolyzed to kynurenine. Indoximod (1-methyl-D-tryptophan or D1MT) is an orally administered IDO inhibitor [78,79]; a Phase I trial (NCT02835729) is ongoing to test indoximod in combination with idarubicin and cytarabine for patients with newly diagnosed AML. Another orally available IDO inhibitor, lirondostat (BMS986205), is in a Phase II clinical trial (NCT02935634) in combination with nivolumab for AML or MDS patients [79].

Nonessential amino acids (NEAAs) can be synthetized by normal cells, and many tumor cells urgently need NEAAs for proliferation and cell activity [80].

Glutamine is the most abundant amino acid in the blood. The enzymes containing glutamine amido-transferase (GATase) domains catalyze the conversation of glutamine to glutamate. Glutamate can be synthetized from glutamine via mitochondrial glutaminases (GLS1 or GLS2). GLSs are significantly overexpressed in AML, and many targeting drugs have been developed. GLS1 and GLS2 allosteric inhibition with telaglenastat (CB-839) significantly reduces intracellular glutamate levels, resulting in a decrease of cell viability [81]. Recent data demonstrated that the treatment with telaglenastat leads to a reduction in mitochondrial respiration and induction of apoptosis in AML cell lines [82]. In addition, the combinatorial administration of telaglenastat with the FLT3 inhibitor (AC220) promotes the sensitivity of AML cells to treatment. The FLT3 inhibitor alters the metabolism in AML cells bearing the FLT3 and IDH mutations away from glucose towards glutamine catabolism [82].

Finally, arginine in a non-essential amino acid whose metabolism is often altered in leukemic cells [80]. Arginine is consumed by AML blasts with T-cell dysfunction, thus creating an immunosuppressive microenvironment [83]. Arginine is metabolized intracellularly by different tissue-specific enzymes, including arginase, which converts arginine to ornithine and urea [84]. Advanced clinical trial Phase I (NCT02903914) is ongoing on arginase 1/2 inhibitor CB-1158, which is orally administrated [85].

### 3.5. Mitophagy Inhibition

Mitophagy is a process by which damaged or dysfunctional mitochondria are selectively targeted for degradation by the autophagy machinery [86]. The dysregulation of mitophagy has been implicated in the pathogenesis of various diseases, including cancer [87]. In leukemia, mitophagy plays a crucial role in regulating the survival and proliferation of leukemic cells, including AML and CLL, by providing energy and metabolic intermediates and contributing to leukemic cell survival [10,88]. Furthermore, mitophagy has been shown to play a role in the drug resistance of leukemia cells that often exhibits increased mitophagy to survive and proliferate even in the presence of cytotoxic drugs [89]. Targeting mitophagy has emerged as a potential and promising therapeutic strategy for the treatment of leukemia since the dysregulation of mitophagy-related proteins, including PINK1, LC3, BNIP3, and Parkin, significantly contribute to the pathogenesis [90].

At present, most mitophagy drugs are not a therapeutical choice since the results of clinical trials are confounding. Nevertheless, we noticed cloroquine (CQ) and bafilomycin A1 (Baf A1), well-known autophagy inhibitors that have shown significant results only in vitro experiments against LSCs, since the toxicity and poor pharmacokinetics have limited their use in clinical practice [91,92]. XRK3F2, another mitophagy inhibitor able to block p62 activity, has demonstrated in patient-derived tumor xenograft (PDX) AML models a selective ability to inhibit LSCs without affecting HSCs [93]. For the above reasons, combination therapies with conventional drugs have been explored to improve treatment outcomes in leukemia and, in some cases, have proven promising efficacy. A Phase I/II clinical trial (NCT01682516), characterized by hydroxychloroquine in combination with azacytidine, showed promising efficacy, while another Phase I/II trial (NCT03250273) is investigating the safety and efficacy of the autophagy inhibitor spautin-1 in combination with chemotherapy in patients with relapsed or refractory acute lymphoblastic leukemia. Moreover, the combination of CQ and Venetoclax, a Bcl-2 inhibitor, has been shown to induce apoptosis in leukemic cells and to improve treatment outcomes in preclinical models [94].

Finally, a different approach of mitophagy inhibition featured the use of specific siRNAs or small molecules against mitophagy-related proteins.

To date, the key targets inactivated with this strategy are the PINK1-Parkin and the Mcl-1pathways. The first interference, by preventing the recognition and tagging of damaged mitochondria, has led to the accumulation of dysfunctional mitochondria, the destruction of cellular metabolism, and, ultimately, to cell death [92]. The second interference, by reducing the level of a crucial anti-apoptotic protein, has induced a marked inhibition of cell survival and proliferation [95,96].

Even if exciting and promising, more studies are needed to fully understand their effectiveness and potential side effects in the context of AML treatment.

## 4. Targeting Bcl-2 Proteins in AML

**Figure 2:**
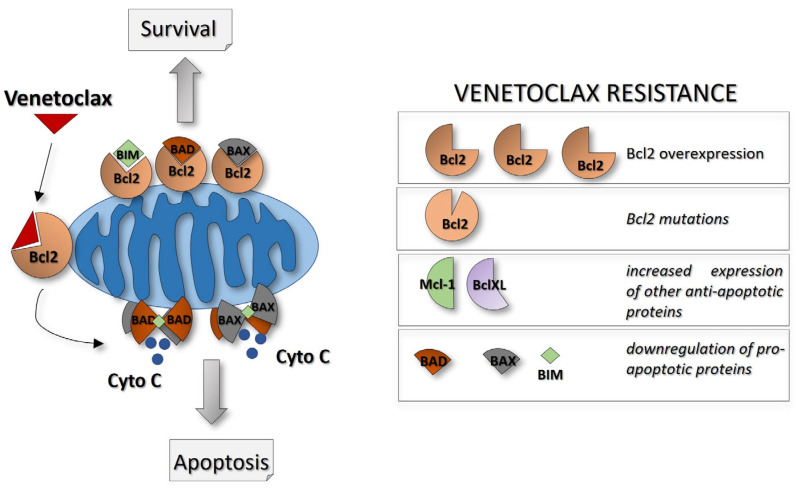
Mechanism of action of Venetoclax. The BH3-mimetic Venetoclax interacts selectively with BCL2 in the BH3-binding grooved pocket, preventing its interaction with the pro-apoptotic proteins Bad and Bax. The main consequence is the release of cytochrome c from the mitochondria, and the formation of pores in the mitochondrial membrane, triggering a cascade of events that ultimately leads to apoptosis. To avoid Venetoclax effects, different forms of resistance may be activated by leukemic cells, including Bcl-2 overexpression, Bcl-2 mutations, or deregulation of other anti-apoptotic proteins. Abbreviations: Cyto C: cytochrome c.

B-cell lymphoma 2 protein (Bcl-2) proteins family collectively orchestrates mitochondrial integrity and modulates apoptotic pathways. Its dysregulation often leads to the survival and proliferation of leukemia cells, contributing to disease progression and resistance to conventional therapy. In this regard, Venetoclax was classified as a selective and oral small-molecule “BH3-mimetic” antagonist of the Bcl-2, since it was designed to specifically mimic and inhibit the BH3 domain (Bcl-2 homology domain 3) of Bcl-2 (Figure 2) [97]. The result is a strong inability of Bcl-2 to bind and neutralize pro-apoptotic proteins BAX and/or BAK and, additionally, a destabilization of H+ gradient across the mitochondrial inner membrane [98]. The consequence is MOMP (mitochondrial outer membrane permeabilization), which results in the release of cytochrome c to the cytoplasm and apoptosis pathway induction. Bcl-2 overexpression has been implicated in several forms of cancer cells that rely heavily on Bcl-2 for their survival, such as chronic lymphocytic leukemia (CLL), certain types of lymphomas, and AML [99,100,101,102]. Therefore, Venetoclax was initially used in CLL cases with 17p loss, where therapeutical options with positive outcome are few [103].

To date, since elevated Bcl-2 expression has been reported in more than 90% of CLL patients, the use of Venetoclax has been extended to relapsed and refractory patients as monotherapy, or in combination with rituximab, obinutuzumab, or ibrutinib [104]. After its approval for CLL treatment, Venetoclax started to be employed in AML, since Bcl-2 expression levels are typically high and are implicated in survival and resistance to conventional therapies of AML cells [105,106,107]. The FDA approved, in 2018, the use of the Venetoclax-combination therapy with hypomethylating agents (HMA) such as azacytidine and decitabine in patients who are not eligible for intensive chemotherapy or stem cell transplant [108]. The azacitidine–Venetoclax combination regimen (VIALE-A trial) demonstrated its superiority compared to azacytidine alone with an increased rate of complete responses and improved overall survival [6].

These promising results extended the use of the Venetoclax–HMA regimen as a front-line treatment instead of an induction chemotherapy for naive unfit AML patients. Several studies confirmed the important role of this regimen even in Refractory/Relapsed (R/R) AML patients [109,110]. Indeed, the Bcl-2 overexpression in LSCs increases their susceptibility to Venetoclax treatment, limiting OXPHOS and amino acid metabolism, resulting in a rapid LSCs eradication without interfering with normal HSCs tools [18,111].

In this scenario, a significant correlation between refractory to Venetoclax and AMLs known for their ability to increase OXPHOS, such as acute monocytic leukemia (AML-M5), has been registered [112]. In addition, patients harboring *FLT3-ITD*, *RAS*, or *TP53* mutations showed a reduction in sensitivity to Venetoclax-based treatment due to their ability to decrease the LSCs Bcl-2 levels (TP53) [113], increase other anti-apoptotic proteins such as BCL-XL (FLT3) [114,115], or activate the use of alternative energy sources such as glycolysis of fatty acid metabolism (RAS) [116]. However, a positive association between NPM1, TET2, and IDH1/2 mutations and increased response rates and OS has been observed. Therefore, resistance to Venetoclax continues to be a critical issue in the treatment of leukemia. Despite the encouraging results, there is a higher percentage of responders to Venetoclax–HMA relapse after a median of about 18 months [117].

Due to its relevance, the biological basis for Venetoclax resistance has been extensively studied. To date we can identify the main mechanisms involved (Figure 2): occurrence of Bcl-2 mutations (e.g., Gly101Va) that cause a reduction in Venetoclax affinity for Bcl-2 [117], upregulation of other anti-apoptotic proteins like BCL-XL or MCL-1, which can provide alternative survival signals for the leukemia cells [118], reduced expression of pro-apoptotic proteins crucial for Venetoclax-induced apoptosis [119], and increased OXPHOS or alternative sources for energy metabolism [120]. To overcome resistance to Venetoclax in leukemia, a variety of approaches are under investigation. New Bcl-2 inhibitors that can target alternative anti-apoptotic proteins, such as MCL-1 or BCL-XL, are being used in clinical trials [121,122,123,124].

Finally, identifying biomarkers (e.g., higher levels of Bcl-2, genetic mutations, minimal residual disease (MRD) after Venetoclax treatment) that can predict which patients are more eligible to develop resistance to Venetoclax is an active area of research that may help to guide treatment decisions [125].

## 5. Conclusions

In conclusions, it is now clear that leukemic stem cells have a peculiar metabolism that can be exploited for inducing a selective death of tumor cells. A better knowledge of the mitochondrial metabolism of the leukemic cells, as well as of the drug-resistance mechanisms that aim to act against these metabolic pathways, could represent the basis for an increasingly personalized and effective therapy.

## Figures and Tables

**Table 1:** Therapeutic approaches for mitochondrial targets in AML. Summary of the most relevant therapies able to modulate mitochondrial activity in AML. The list also included some mechanisms related to autophagy inhibition, a process that is not directly related to mitochondria but crucial for metabolic processes of AML cells. Abbreviations: FDA: federal drug administration; SAHH: S-adenosyl homocysteine hydrolase; IDH1/2: isocitrate dehydrogenase 1/2; IDO: indoleamine 2,3-dioxygenase; PRMT5: protein arginine methyltransferase 5; HER2: Human epidermal growth factor receptor-2; MaT2A: methionine adenosyltransferase 2A; FLT3: FMS-like tyrosine kinase 3; GLSs: glutaminases; Bcl-2: B-cell lymphoma 2; SDH: succinate dehydrogenase; and p62: ubiquitin conjugating enzyme E2-N and sequestosome-1.

Name	Code	Mechanism	Status
Arsenic Trioxide + All-trans Retinoic Acid	ATO/ATRA	Stimulation of promyelocytic cells differentiation	FDA approved
Bafilomycin A1 (Baf A1)	CAS 88899-55-2	Inhibition of autophagic flux	FDA approved
Chloroquine (CQ)	P01BA01	Inhibition of autophagosome-lysosome fusion	FDA approved
Chloroquine + Venetoclax		Induction of cancer cell death	Preclinical studies
DZ2002	DZ2002	SAHH inhibitor	
Enasidenib	AG-221	IDH2 inhibitor	FDA approved
Hydroxychloroquine + Azacytidine	NCT01682516	DNA methyltransferase inhibition and autophagy inhibition	Phase I/II clinical trial
Indoximod	NLG-8189	IDO inhibitor	
Indoximod + Idarubicin + Cytarabine	NCT02835729	IDO inhibition and DNA topoisomerase and DNA polymerase inhibition	Phase I clinical trial
Ivosidenib	AG-120	IDH1 inhibitor	FDA approved
Linrodostat	BMS-986205	IDO1 inhibitor	Phase III clinical trial
Linrodostat + Nivulimab	NCT02658890	IDO inhibition	Phase I/II clinical trial
LLY-283	LLY-283	PRMT5 inhibitor	Preclinical studies
Mubritinib	TAK-165	HER2 inhibitor	FDA approved
Numidargistat	CB-1158 (NCT02903914)	Arginase inhibitor	Phase I clinical trial
PF-9366	PF-9366	Mat2A inhibitor	Preclinical studies
Quizartinib	AC-220	FLT3 inhibitor	Under approval
Spautin-1	Spautin-1	Autophagy inhibitor	Phase I/II clinical trial
Telaglenastat	CB-839	GLSs inhibitor	
Venetoclax	L01XX52	Bcl-2 inhibitor	FDA approved
Venetoclax + Azacytydine	NCT02993523	Inhibition of SDH glutathionylation	Phase III clinical trial
XRK3F2	XRK3F2	P62–ZZ inhibitor	Preclinical studies
Zalcitabine	ddC	Mitochondrial DNA polymerase inhibitor	FDA approved

## Data Availability

No new data were created or analyzed in this study. Data sharing is not applicable to this article.
